# Intestinal microbiota of preterm infants differ over time and between hospitals

**DOI:** 10.1186/2049-2618-2-36

**Published:** 2014-10-01

**Authors:** Diana H Taft, Namasivayam Ambalavanan, Kurt R Schibler, Zhuoteng Yu, David S Newburg, Doyle V Ward, Ardythe L Morrow

**Affiliations:** 1 Department Pediatrics, Perinatal Institute, Cincinnati Children’s Hospital Medical Center, 3333 Burnet Ave., MLC 7009, Cincinnati, OH 45229, USA; 2 Department of Environmental Health, University of Cincinnati College of Medicine, 3223 Eden Ave., Cincinnati, OH 45267, USA; 3 Department of Pediatrics, University of Alabama at Birmingham, 1700 6th Ave. S, 9380 176F WIC, Birmingham, AL 35249, USA; 4 Department of Biology, Boston College, Higgins Hall, 140 Commonwealth Ave., Chestnut Hill, MA 02467, USA; 5 Broad Institute, 415 Main St., Cambridge, MA 02141, USA; 6 Division of Biostatistics and Epidemiology, Cincinnati Children’s Hospital Medical Center, 3333 Burnet Ave., Cincinnati, OH 45229, USA

**Keywords:** Infants, Premature, Microbiome, Geo-temporal analysis, Microbial succession

## Abstract

**Background:**

Intestinal microbiota are implicated in risk of necrotizing enterocolitis (NEC) and sepsis, major diseases of preterm infants in neonatal intensive care units (NICUs). Rates of these diseases vary over time and between NICUs, but time and NICU comparisons of the intestinal microbiota of preterm infants are lacking.

**Methods:**

We included 66 singleton infants <29 weeks gestational age with stool samples collected between postnatal days 3 to 21 of life who survived free of NEC and sepsis. Infants were enrolled during 2010 and 2011. Twenty-six infants were enrolled at hospital 1 in Cincinnati, OH, and 40 infants were enrolled at hospital 2 in Birmingham, AL. Samples collected from days 3–9 (“week 1”) and days 10–16 (“week 2”) were compared between years and hospitals. Microbial succession was compared between hospitals in 28 infants with samples from the first 3 weeks of life. DNA extracted from stool was used to sequence the 16S rRNA gene by Illumina MiSeq using universal primers. Resulting operational taxonomic unit tables were analyzed for differences between years and hospitals using linear discriminant analysis effect size algorithm (LEfSe; significance, *p* < 0.05).

**Results:**

Significant variation was observed in infant microbiota by year and hospital. Among hospital 1 infants, week 1 samples had more phylum *Firmicutes* (class *Bacilli*, families *Clostridiaceae* and *Enterococcaceae*) in 2010 and more phylum *Proteobacteria* (family *Enterobacteriaceae*) in 2011; week 2 samples did not significantly vary over time. However, among hospital 2 infants, the week 1 shift was nearly opposite, with more *Proteobacteria* (*Enterobacteriaceae*) in 2010 and more *Firmicutes* (*Bacilli*) in 2011; week 2 samples exhibited the same pattern. Regression analysis of clinical covariates found that antibiotic use had an important influence but did not explain these observed shifts in microbiota over time and between hospitals. Microbial succession also differed by hospital, with greater change in microbiota in hospital 1 than hospital 2 infants (*p* < 0.01, Jaccard distance).

**Conclusion:**

Colonizing microbiota differ over time and between NICUs in ways that could be relevant to disease. Multi-site, longitudinal studies are needed to reliably define the impact of intestinal microbiota on adverse outcomes of preterm infants.

## Background

John Snow’s investigation of cholera in 1854 pioneered the understanding of disease risk in relation to not only individual characteristics (person) but also geographic patterns (place) and temporal patterns (time) [1]. Since then, epidemiologic investigations have advanced our understanding of the carriage frequency, pathogenicity, and transmission dynamics of enteric organisms in relation to person, place, and time. With the advent of high-throughput sequencing methods, research on the human intestinal microbiome has led to important insights regarding the impact of personal characteristics such as age, gender, diet, and disease state on the colonizing microbial communities. By comparison, few studies have investigated geographic and temporal influences on human microbial communities. Understanding temporal and geographic influences on the colonizing human microbiota is a critical step towards understanding the extent to which studies undertaken in a population defined by time and location can be generalized to the same population at a later time or populations at other locations.

Geographic differences in the human intestinal microbiota have been more extensively studied than temporal differences within populations. For example, *Bacteroidetes* were shown to dominate the microbiomes of African children, while *Firmicutes* and *Proteobacteria* were enriched in the microbiota of European children [2]. Similarly, Lee et al. reported differences in the microbiota of Korean and US twins, particularly in taxa belonging to *Firmicutes* and *Bacteroidetes*[3]. In other comparative studies, the microbiota of built environments can differ significantly [4]; such differences could be attributed to the broader local environment or to the inhabitants of the built environment. A study of the colonization patterns of preterm infants in three distinct Florida neonatal intensive care units (NICUs) demonstrated significant differences in the relative abundance of the phyla *Bacteroidetes*, *Proteobacteria*, and *Firmicutes* in the inhabitants of these NICUs [5]. Variations in infant microbiota can lead to differences in environmental exposures and vice versa, as bacteria in the NICU environment are also found to colonize the preterm infant gut [6].

It is not known whether colonization patterns of intestinal microbial communities shift within human populations over calendar time, but temporal patterns are well documented with many enteric pathogens [7]. Cholera outbreaks have one or two annual peaks, in spring and fall. *Helicobacter pylori* also shows peak colonization in children in spring and fall [8,9]. Although it is unclear if commensals also exhibit such temporal patterns, one study found that the microflora of buildings varied over a single year of sampling, and in one of the studied buildings, there was a shift from *Actinobacteria* to *Proteobacteria* during the year [4].

To address the question of temporal and geographic differences in the intestinal microbiota, we conducted a prospective study of the intestinal microbiota of preterm infants at two large, geographically separated level III NICUs. We used 2 years of data from this cohort study to test the hypothesis that initial microbiota colonization of disease-free survivors changes over time and differs between birth hospitals.

## Results

### Subjects

16S rDNA sequence data were generated from a total of 66 infants with 51 samples in week 1 (22 from hospital 1 and 29 from hospital 2, both years) and 60 samples in week 2 (24 from hospital 1 and 36 from hospital 2, both years). A total of 28 infants (18 from hospital 1 and 10 from hospital 2, both years) had samples available from all 3 weeks for use in the analysis of microbial succession. The infant characteristics by hospital and by week are presented in Table 1. Infants at both hospitals were generally well matched demographically in week 1 and week 2, with more black infants born and more infants born to slightly younger mothers at hospital 2. Infant characteristics within a single hospital in weeks 1 and 2 between 2010 and 2011 were similar with the exception that hospital 2 mothers were younger in 2010 than those in 2011 (weeks 1 and 2, *p* < 0.05, data not shown). Similar types of antibiotics were used at both hospitals, a combination of ampicillin and gentamicin for the majority of infants. Infants at hospital 2 were also more likely to be exposed to antibiotics and for longer durations in the first 14 days of life. Also, a higher percentage of mothers received antibiotics at the time of delivery at hospital 2. At hospital 1, three infants received no antibiotics and one infant received nafcillin and gentamycin. At hospital 2, eight infants received ampicillin and tobramycin. The first day of life of enteral feeding was similar at both hospitals, but the hospitals differed in their use of formula. Hospital 1 infants received exclusively either mother’s own milk or pasteurized human donor milk for the first 14 days of life, while approximately 20% of hospital 2 infants were fed formula (Table 1).

**Table 1:** Characteristics of infants with samples included in analysis, by week

	**Hospital 1, week 1**		**Hospital 2, week 1**		**Hospital 1, week 2**		**Hospital 2, week 2**	
	**2010**	**2011**	**2010**	**2011**	**2010**	**2011**	**2010**	**2011**
	***n*** **= 9**	***n*** **= 13**	***n*** **= 12**	***n*** **= 17**	***n*** **= 11**	***n*** **= 13**	***n*** **= 15**	***n*** **= 21**
Birthweight, mean ± sd (g)	963 ± 216	965 ± 261	938 ± 234	953 ± 223	985 ± 193	995 ± 261	914 ± 216	962 ± 207
Birth length, mean ± sd (cm)	35.8 ± 3.0	35.2 ± 2.9	34.0 ± 3.1	34.9 ± 2.9	35.8 ± 2.7	35.5 ± 2.9	33.7 ± 3.5	36.3 ± 6.9
GA at birth, weeks (25th–75th percentile)	26 (26–28)	28 (26–28)	27 (26.8–28)	27 (26–28)	26 (25.5–27.5)	28 (26–28)	27 (26–28)	27 (26–28)
Male (%)	5 (56%)	6 (46%)	5 (42%)	7 (41%)	4 (36%)	7 (54%)	6 (40%)	11 (52%)
Hispanic (%)	0 (0%)	1 (7.7%)	1 (8.3%)	0 (0%)	0 (0%)	1 (7.7%)	1 (6.7%)	0 (0%)
Black (%)	5 (56%)	6 (46%)	8 (67%)	6 (35%)	3 (27%)	2 (15%)	10 (67%)	8 (38%)
Cesarean section (%)	5 (56%)	5 (38%)	6 (50%)	10 (59%)	6 (55%)	6 (46%)	7 (47%)	10 (48%)
Maternal abx at delivery (%)	6 (67%)	10 (77%)	11 (92%)	17 (100%)	8 (73%)	10 (77%)	14 (93%)	20 (95%)
Maternal age ± sd (years)^a^	27.4 ± 1.6	28.0 ± 1.4	**22.5 ± 4.9**	**x27.8 ± 6.1**	28.5 ± 6.7	29.1 ± 5.6	**21.9 ± 5.1**	**27.7 ± 5.7**
Maternal parity (25th–75th percentile)	2 (1–3)	1 (1–3)	2 (1–3)	2 (1–2)	2 (1.5–3)	1 (1–3)	2 (1–3)	1 (1–3)
Preeclampsia (%)	1 (11%)	2 (15%)	3 (25%)	1 (5.8%)	1 (9.1%)	3 (2.3%)	3 (20%)	1 (4.8%)
Day of life of sample collection, median (25th–75th percentile)^b^	7 (5–7)	6 (5–8)	**6 (5.8–6.3)**	**8 (7–8)**	13 (13–14)	14 (11–14)	11 (10–12)	10 (10–12)
Days on abx in the first 7 days ± sd	4.0 ± 2.6	3.1 ± 2.1	4.7 ± 2.0	3.8 ± 1.7	NA	NA	NA	NA
Days on abx in the first 14 days ± sd	NA	NA	NA	NA	4.4 ± 3.1	3.6 ± 3.8	7.6 ± 4.4	5.4 ± 3.4
Days of life of first enteral feed, mean ± sd	4.6 ± 3.7	2.5 ± 0.66	3 ± 1.3	2.8 ± 1.2	3.6 ± 1.5	2.7 ± 0.75	3.6 ± 2.4	3 ± 1.6
Median percent formula feeds in the first 7 days (25th–75th percentile)	0% (0%, 0%)	0% (0%, 0%)	0% (0%, 35.7%)	0% (0%, 25%)	NA	NA	NA	NA
Median percent formula feeds in the first 14 days (25th–75th percentile)	NA	NA	NA	NA	0% (0%, 0%)	0% (0%, 0%)	8.3% (0%, 31.7%)	6.7% (0%, 18.2%)

^a^Significantly different between years in hospital 2 week 1, *p* = 0.015, and hospital 2 week 2, *p* = 0.003; ^b^significantly different between years in hospital 2 week 1, *p* < 0.01. Data in bold are values which are significantly different.

### Microbial diversity

**Figure 1:**
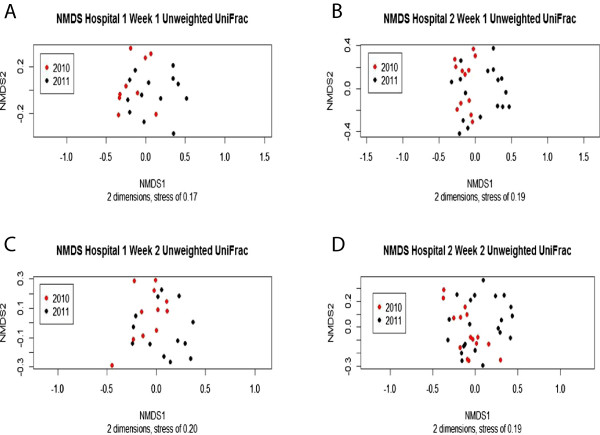
**NMDS ordination of week 1 and week 2 samples for each hospital based on unweighted UniFrac.** Samples from 2010 are indicated as *red dots* and samples from 2011 are indicated as *black dots*. In each ordination, the 2010 samples (*red dots*) tend to be more on the left side while the 2011 samples (*black dots*) tend to be more on the right suggesting a shift in microbial community composition between the years. **(A)** Week 1 results from hospital 1. Number of dimensions was 2 and stress was 0.17. **(B)** Week 1 results from hospital 2. Number of dimensions was 2 and stress was 0.19. **(C)** Week 2 results from hospital 1. Number of dimensions was 2 and stress was 0.20. **(D)** Week 2 results from hospital 2. Number of dimensions was 2 and stress was 0.19.

We observed no significant differences in alpha diversity in week 1 or week 2 samples, by hospital or year, using Simpson or Chao1 metrics. Furthermore, clear separation between hospitals or years was not observed in weighted ordinations (not shown). However, unweighted UniFrac ordinations stratified by hospital showed a trend towards separation based on year (Figure 1). Unweighted UniFrac is a distance measure that uses the presence/absence of operational taxonomic units (OTUs) and their phylogenetic relatedness, while weighted UniFrac additionally considers relative abundance of the OTUs. The difference in the unweighted UniFrac ordination and lack of difference in the weighted UniFrac ordination suggest that there are significant differences between years in the OTUs present in the two hospitals but that the taxa comprising the majority of the reads are similar between years.

**Figure 2:**
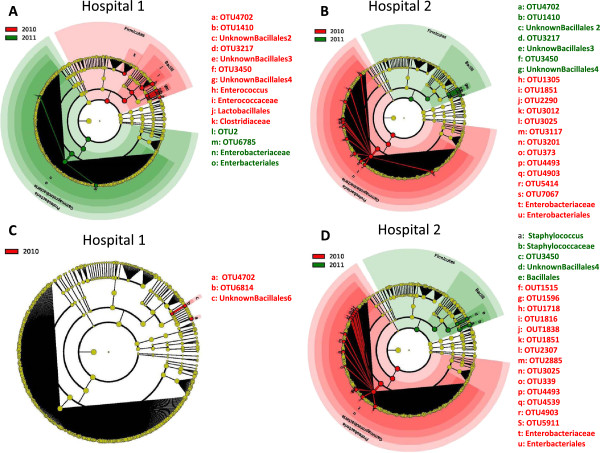
**Cladograms generated by LEfSe indicating differences in taxa between hospitals in samples from weeks 1 and 2.** The *central yellow dot* in each cladogram represents kingdom; each *successive circle* is one step lower phylogenetically (phylum, class, order, family, and OTU). *Regions in red* indicate taxa that were enriched in 2010 compared to those in 2011, while *regions in green* indicate taxa that were enriched in 2011 compared to those in 2010. **(A)** Week 1 sample from hospital 1. In 2010, samples were enriched for *Firmicutes*, with a shift in microbiota towards an increase in *Proteobacteria* in 2011. **(B)** Week 1 sample from hospital 2. In 2010, infants at hospital 2 were enriched in *Proteobacteria*, while in 2011, they were enriched in *Firmicutes.***(C)** Week 2 sample from hospital 1. Only OTU level differences were detectable between 2010 and 2011. **(D)** Week 2 sample from hospital 2. In 2010, samples were enriched for *Proteobacteria*. In 2011, the infant microbiota were enriched in *Firmicutes*, particularly, in *Bacilli*.

In week 1 samples, comparison of infants within each hospital by year revealed that for hospital 1, the 2010 infants were significantly enriched in *Firmicutes* compared to 2011 infants, while the 2011 infants were enriched in *Proteobacteria* compared to 2010 infants (Figure 2A)*.* Differences also occurred at lower phylogenetic levels between years, with *Clostridiaceae* and *Enterococcaceae* (families of phylum *Firmicutes*) enriched in 2010. In 2011, infants were enriched in *Enterobacteriaceae*, the largest family of phylum *Proteobacteria.* In week 1 samples of hospital 2, infants showed the opposite pattern by year on the phylum level, such that the 2010 infants were enriched in *Proteobacteria* while the 2011 infants were enriched in *Firmicutes* (Figure 2B). Hospital 2 infants also showed differences at lower phylogenetic levels. In 2010, they were enriched in family *Enterobacteriaceae*, while in 2011, they were enriched in OTUs of class *Bacilli*. As a correlate of these shifts within hospitals, in 2010, the infant microbiota differed between hospitals with hospital 1 infants having more *Firmicutes* and hospital 2 infants having more *Proteobacteria*. In 2011, infants from both hospitals had similar microbiota, with only OTU level differences detected (not shown).

In week 2 samples, comparing infants within hospital 1 by year revealed that colonization patterns were relatively stable between 2010 and 2011 demonstrating only OTU level differences (Figure 2C). In hospital 2 infants, colonization patterns changed between years with decreased *Proteobacteria* and increased *Firmicutes* in 2011 (Figure 2D). There were also differences at lower phylogenetic levels in hospital 2, with infants in 2010 enriched in *Staphylococcaceae* and infants in 2011 enriched in *Enterobacteriaceae*. Week 2 samples were different between the two hospitals, with hospital 2 enriched in *Proteobacteria* and hospital 1 enriched in *Firmicutes*. In 2011, the week 2 infant colonization patterns were similar between the hospitals, consistent with the observation of week 1 colonization patterns.

**Figure 3:**
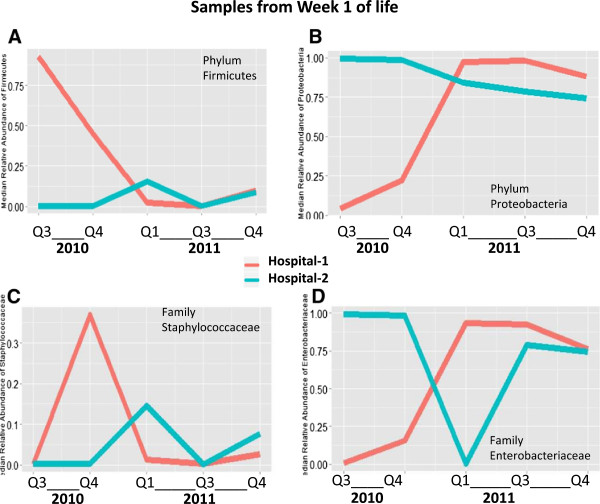
**Median relative abundance of phyla *Firmicutes *and *Proteobacteria *by quarter in week 1 sample.** Line plots indicating shifts in the median relative abundance of selected taxa by quarter for hospital 1 and hospital 2 in week 1. *Red lines* indicate hospital 1 and *blue lines* indicate hospital 2. Quarters 1, 7, and 8 are excluded because fewer than three infants per hospital had samples during those quarters. **(A)** Median relative abundance of *Firmicutes*. In hospital 1, *Firmicutes* started high in 2010 and then fell in 2011. In hospital 2, *Firmicutes* started low in 2010 and then rose slightly in 2011. **(B)** Median relative abundance of *Proteobacteria*. At both sites, *Proteobacteria* followed a pattern opposite to that of *Firmicutes.***(C)** Median relative abundance of *Staphylococcaceae*, the most commonly detected family of *Firmicutes*. Pattern in the relative abundance of *Staphylococcaceae* was similar to that of *Firmicutes* for most quarters. **(D)** Median relative abundance of *Enterobacteriaceae*, the most commonly detected family of *Proteobacteria*. Pattern in relative abundance of *Enterobacteriaceae* was similar to that of *Proteobacteria* for most quarters.

To examine the changes in infant colonization over time in greater detail, we plotted the median relative abundance of phyla *Firmicutes* and *Proteobacteria* by quarter in weeks 1 and 2 (Figure 3). Quarters with fewer than three study infants enrolled from each hospital were excluded from the graph. In week 1 samples, hospital 1 infants showed a sharp decline over time in median *Firmicutes* abundance and a corresponding increase in *Proteobacteria* abundance; hospital 2 infants showed fluctuating amounts of *Firmicutes* and a modest decline in *Proteobacteria* colonization (Figure 3A,B). We then examined the median relative abundance of *Staphylococcaceae*, the most abundant family in phylum *Firmicutes*, and the median relative abundance of *Enterobacteriaceae*, the most abundant family in phylum *Proteobacteria* (Figure 3C,D). These major bacterial families similarly demonstrated time trends, though differed from the patterns of their respective phyla in a few quarters.

**Figure 4:**
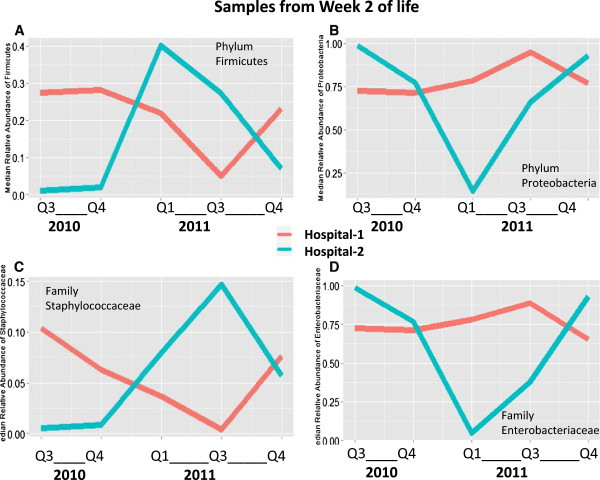
**Median relative abundance of phyla *Firmicutes *and *Proteobacteria *by quarter in week 2 sample.** Line plots indicating shifts in the median relative abundance of selected taxa by quarter for hospital 1 and hospital 2. *Red lines* indicate hospital 1 and blue lines indicate hospital 2. Quarters 1, 7, and 8 are excluded because fewer than three infants per hospital had samples during those quarters. **(A)** Median relative abundance of *Firmicutes*. In hospital 1, *Firmicutes* again began high in 2010 and then fell in 2011 before rebounding slightly. In hospital 2, relative abundance of *Firmicutes* peaked in early 2011. **(B)** Median relative abundance of *Proteobacteria*. Relative abundance of *Proteobacteria* appears to be inversely related to relative abundance of *Firmicutes*. **(C)** Median relative abundance of *Staphylococcaceae*, the most commonly detected family of *Firmicutes*. The abundance of *Staphylococcaceae* follows the same general trend as *Firmicutes*. **(D)** Median relative abundance of *Enterobacteriaceae*, the most commonly detected family of *Proteobacteria*. The relative abundance of *Enterobacteriaceae* follows the same general trend as *Proteobacteria*.

Regarding time trends by quarter in week 2 samples, infants at hospital 1 again showed fluctuations in *Firmicutes* and *Proteobacteria* relative abundance, but different from week 1 sample, there was no clear distinction between study years. Similar to week 1 sample, infants at hospital 2 showed a peak in *Firmicutes* and a drop in *Proteobacteria* during the second quarter of 2011, likely explaining the difference in the abundance of these phyla between the 2 years in week 2 (Figure 4A,B). For most quarters, changes in the relative abundance of *Staphylococcaceae* again corresponded with changes in the relative abundance of *Firmicutes* and changes in the relative abundance of *Enterobacteriaceae* again corresponded with changes in the relative abundance of *Proteobacteria* (Figure 4C,D).

To determine the influence of differences in clinical or methodologic factors on the differences observed over study years within each NICU, we modeled the relative abundance of *Proteobacteria* using generalized estimating equations (GEE) including samples from both weeks 1 and 2 (Table 2). *Proteobacteria* was examined as an outcome using two different models: first, as a continuous variable in a linear model and second, in a logistic model with the relative abundance of *Proteobacteria* dichotomized into high versus low relative abundance based on a natural cut-point in the data distribution. The high category was defined as more than 25% relative abundance of *Proteobacteria.* Across both hospitals and years, 74% of samples in week 1 and 60% of samples in week 2 were classified as high *Proteobacteria.*

**Table 2:** **Results of GEE models of *Proteobacteria *** **relative abundance for weeks 1 and 2 sample within each hospital NICU**

**Predictor variable**	**Hospital 1**		**Hospital 2**	
	**Coefficient**	***p *** **value**	**Coefficient**	***p *** **value**
Linear models				
Year 2011 vs. 2010	*0.313*	*0.003*	*-0.241*	*0.020*
Maternal antibiotic use			0.236	0.063
Infant gestational age			*-0.0863*	*0.0082*
Logistic models^a^				
Year 2011 vs. 2010	*1.63*	*0.033*	*-2.77*	*0.039*
Maternal age at delivery			0.0853	0.28
Infant gestational age			-0.999	0.11

^a^Logistic models compare odds of high *Proteobacteria* (relative abundance ≥25%) vs. low *Proteobacteria*.

Models were hospital specific. In both hospitals, models included the following covariates: birth year, maternal antibiotic use, infant antibiotic use in the first 14 days of life, infant gestational age, maternal age at delivery, delivery mode, and day of sample collection. Percentage of feeds that were formula was not included in the hospital 1 model because infants in that hospital received no formula prior to day of life 14. In hospital 1, whether or not a sample was stored in thioglycollate was included to account for any storage protocol differences, as all week 1 samples and 19 (80%) of 24 samples in week 2 were stored in thioglycollate. In hospital 2, we also analyzed the percentage of feeds that were formula; we did not analyze that variable in hospital 1, where all infants were human milk fed. Backwards elimination was used to determine the final model. Any variable with a *p* value greater than 0.10 that did not change the coefficient of the year variable by more than 10% was eliminated starting with the covariate with the highest *p* value. Both models at both hospitals found that birth year remained significant, but the other covariates dropped out of most models, as shown in Table 2.

**Figure 5:**
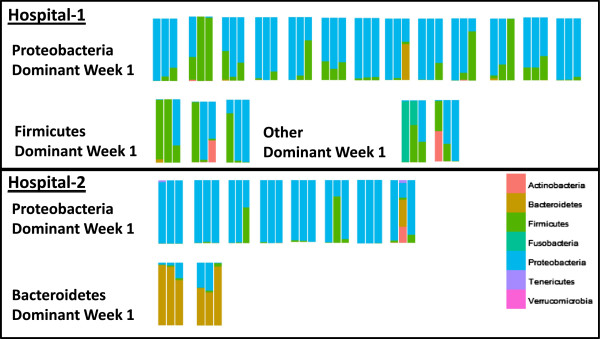
**Changes in relative abundance of phyla by week for infants with samples collected in weeks 1, 2, and 3.***Each set of three bars* represents samples from a single infant with samples from week 1, then week 2, and finally week 3 going from left to right. Samples are grouped by hospital and dominant phyla (>50% of reads) in week 1. The other category also contains an infant who had no single phyla accounting for >50% of the reads. Infants from Birmingham had significantly more similar stool samples from week to week compared to infants from Cincinnati as measured by Jaccard distance calculated at the OTU level and tested by Kruskal-Wallis (*p* < 0.01).

We then analyzed microbial succession patterns within each hospital over the first 3 weeks of life. There were no significant differences in Jaccard index between repeated infant samples in infants born in 2010 compared to infants born in 2011 at hospital 1. Since there was only a single infant with sample from all 3 weeks in 2011, a similar comparison by time could not be conducted. Infants were therefore included in a single analysis comparing the hospitals regardless of birth year. At both hospital 1 and hospital 2, most infants had samples in week 1 of life with *Proteobacteria* accounting for >50% of the reads (distribution of infants by quarter was not even; more infants included in this analysis were born in quarters with higher relative abundance of *Proteobacteria* during week 1). At hospital 1, infant samples that were not dominated by *Proteobacteria* were instead dominated by either *Firmicutes* or *Fusobacteria* or lacked any single phyla that accounted for more than half the reads. At hospital 2, however, infant samples that were not dominated by *Proteobacteria* were instead dominated by *Bacteroidetes*. The microbial dominance pattern identified in hospital 2 infants appeared to persist between weeks, but hospital 1 infant microbiota appeared to shift between weeks (Figure 5).

Comparing the Jaccard index calculated from OTU tables confirmed this difference; the Jaccard index between week 1 and week 2, week 1 and week 3, and week 2 and week 3 samples was significantly lower for hospital 2 infants (Kruskal-Wallis, *p* < 0.01 for each of the three time points). A lower value for the Jaccard index indicates a greater degree of similarity between samples, indicating that infants at hospital 2 experienced less change in microbial colonization from week to week than did infants at hospital 1. As change in microbiota between weeks could be affected by duration of the interval of measurement, we examined the days of collection and time interval between samples for the 28 infants in this analysis. The mean day at the time of sample collection by hospital was the same for weeks 1 and 3 but differed in week 2 (week 1—6.4 for hospital 1 and 6.2 for hospital 2, week 2—12.9 for hospital 1 and 11.1 for hospital 2 [*p* < 0.05], and week 3—20.2 for hospital 1 and 20.4 for hospital 2). The mean number of days between sample collections was significantly different between proximal weeks but the same for week 1 to week 3 (the days between week 1 and week 2 samples were 6.5 days for hospital 1 and 4.9 days for hospital 2 [*p* < 0.05], between week 2 to week 3 samples were 7.7 days for hospital 1 and 9.2 days for hospital 2 [*p* < 0.05], and between week 1 to week 3 samples were 14.2 days for hospital 1 and 14.1 days for hospital 2). Since a greater rate of change in microbiota was observed between weeks in hospital 1 compared to hospital 2 for each time interval regardless of whether the time interval was shorter, longer, or the same, we conclude that hospital 1 infants experienced a more rapid turnover in bacteria and that this was not confounded by sample collection timing.

## Discussion

Consistent with previous reports, the microbiota of the preterm infants in our study were most frequently dominated by the phylum *Proteobacteria*; *Firmicutes* was the next most frequent phylum. Nevertheless, our observations support the hypothesis that temporal and geographic factors influence the intestinal microbial colonization of preterm infants. These temporal and geographic differences were not explained by factors known to influence the intestinal microbiome, such as antibiotics. Differences in *Proteobacteria* colonization between 2010 and 2011 remained even after modeling to adjust for the effect of antibiotic use on colonization patterns. Microbial shifts observed at the phylum level involved multiple taxa. Differences within NICUs over time of phylum *Firmicutes* largely included organisms of the class *Bacilli*, and within hospital 1, families *Enterococcaceae* and *Clostridiaceae*, while differences within NICUs over time of phylum *Proteobacteria* largely involved organisms of the family *Enterobacteriaceae*.

Differences in hospital environments over time could be due to changes in environmental management, clinical practices, ongoing quality improvement initiatives, patient population, or other factors. Hospital 1 has a necrotizing enterocolitis (NEC) task force which implemented changes to the standard of care in its NICU over the course of this study to reduce the NEC incidence rate. Changes included the introduction of a standard feeding protocol and use of donor milk when mother’s own milk is unavailable, both of which could potentially influence the developing microbiome of the infant. In February 2010, the hospital 2 NICU moved to a new location. Part of the change in infant colonization during this time could have resulted from the change in location and its evolving microflora. While we report these events as qualitative background information, our study was not designed to analyze the impact of specific events quantitatively.

We speculate that the observed patterns could be due to shifts in the microbiota of the NICU environment itself. Longer-term studies are needed to determine the extent to which temporal shifts in colonization recur in regular patterns, occur by the introduction of specific organisms, or are driven by changes in human behavior or the built environment. Studies with concurrent sampling of both infants and the hospital environment are needed to determine the extent to which hospital resident microbes influence infant colonization patterns and the changes in infant colonization patterns over time.

This report presents the microbial colonization patterns of relatively healthy infants and does not address disease outcomes *per se*. Nevertheless, the differences that we observed could have important clinical consequences. During the period of this study, the hospital NICUs varied in their rates of late-onset sepsis and NEC, two conditions in which intestinal microbiota are implicated. The NICU of hospital 2 had double the rate of late-onset sepsis observed at hospital 1, though sepsis rates remained constant between 2010 and 2011. Rates of NEC at hospital 1 were approximately double those at hospital 2 in 2010, but in 2011, the rates in the two hospitals were indistinguishable.

Nationally, 10% of preterm infants born <29 weeks gestational age develop NEC. This risk has been attributed in part to immature response to LPS-bearing organisms, specifically, excessive TLR4 signaling and hyper-inflammatory response to *Proteobacteria*. Investigators have noted that a surge or “bloom” in *Proteobacteria* (typically, *Enterobacteriaceae*) or a decline in *Firmicutes* precedes NEC [10-12]. The tendency towards surge in *Proteobacteria* and equivalent decline in *Firmicutes* may differ between NICUs. Indeed, in our study, in addition to observing differences between intestinal colonization between the hospitals by week of life, we also observed differences in microbial succession within infants over the first few weeks of postnatal life. Hospital 1 infants experienced a more rapid turnover of bacterial OTUs than did hospital 2 infants. This could have important implications for disease risk.

This study has several limitations: This study was a secondary analysis of a dataset collected for analysis of microbiota prior to onset of NEC. Data from both hospitals and years was available only for the first 2 weeks of postnatal life for most infants; thus, our comparison of hospitals is restricted to those weeks, and we examined microbial succession in the subset of infants with all 3 weeks of data available. Variation in storage protocol due to logistical challenges in the study resulted in use of thioglycollate in one hospital and not the other, which reduced our ability to confidently identify differences in *Bacteroidetes*, *Propionibacteriaceae*, and *Leuconostocaceae*, which are relatively of low abundance in preterm infants. While this methodologic variation is unfortunate, it did not impair the analysis of major taxa in this study. Furthermore, as in all epidemiologic studies, the findings of this study are susceptible to confounding over time or between hospitals. However, we found that the clinical characteristics of infants were generally well matched between hospitals and years, and modeling found that study years within hospitals remained the strongest predictor of the relative abundance of the *Proteobacteria* after controlling for potential confounding by clinical variables or methodologic factors.

## Conclusions

Time of birth and hospital correlate with distinct premature infant intestinal colonization patterns. Identifying these differences in various institutions and over years may provide a biomarker for monitoring disease risk. Our data provide additional evidence for caution regarding the degree to which studies of the microbiome and diseases of prematurity can be generalized to other hospitals or even to the same hospital later in time. Indeed, our data supports the need for large, multi-site, multi-year epidemiological studies in preterm infants and other patient populations to understand the association between intestinal microbial colonization and disease risk. The cohort study from which infants were selected for this study contained too few subjects with NEC and sepsis to effectively assess temporal changes in case infants. This study also clearly indicates a need for careful matching strategies on time and site to examine disease risk in studies of the intestinal microbiota.

### Data availability

Data is available on NCBI SRA, Accession PRJNA63661. Additional metadata and the metadata dictionary are available as a supplementary file to this article (see Additional file 1). This file contains all the metadata used in this article. The OTU table and taxa assignments after rarefaction are also available as supplementary file to this article (see Additional file 2).

## Methods

### Subjects

Study infants were a subset of the infants enrolled from one level III NICU in Cincinnati, OH (hospital 1), and one level III NICU in Birmingham, AL (hospital 2), in 2010 and 2011 as part of an ongoing cohort study of novel biomarkers for NEC. Study infants were ≤32 weeks gestational age. The average daily census of the NICU in hospital 1 was 46 with a capacity of 60, and the average daily census of the NICU at hospital 2 was 85 with a capacity of 120. From the overall cohort, this study was restricted to infants <29 weeks gestational age, singleton births, who survived free of proven NEC or proven sepsis until discharge, and were free of congenital anomalies. These inclusion criteria were applied to establish fundamental comparability between the two NICUs studied and remove the influence of disease outcomes *per se* or other potentially extraneous differences. In addition, all infants had at least one stool sample from week 1 or week 2 that was successfully extracted and sequenced using the methods described below. The Institutional Review Boards at Cincinnati Children’s Hospital and the two participating hospitals approved the study. Parent or guardian consent was obtained for all infants included in this study.

### Sample collection and storage

Stool samples were collected from infants during the first weeks of life on postnatal days 5, 8, 11, 14, and 21 plus or minus 2 days. Due to intermittency of infant stooling, we categorized samples as having been collected in collection windows defined as 3–9 days of life (week 1), 10–16 days of life (week 2), and 17–23 days of life (week 3) in order to optimize the number of infant samples included for analysis at each time point. These time windows were selected because no study infant stooled prior to day of life 3, which is typical for premature infants [13]. Samples were collected from soiled diapers, immediately refrigerated in the NICU, and transported to the laboratory where they remained in the refrigerator until processing for storage at -80°C. Cryogenic storage in hospital 1 utilized thioglycollate buffer except for collection day 11, which was biobanked without buffer to provide a sample for metabolomic analysis. However, when other samples from the same week were not available, day 11 sample was used for 16S analysis. Cryogenic storage in hospital 2 did not use thioglycollate or any other storage buffer.

To determine the impact of thioglycollate buffer on the microbiome, we compared samples from hospital 1 in 2009—seven stored without and seven stored with thioglycollate—matched on day of life of collection and infant clinical history. We found that samples stored without thioglycollate were enriched in *Bacteroidetes*, *Leuconostocaceae*, and *Propionibacteriaceae.* As samples from the hospital 2 NICU were stored without thioglycollate and samples from the hospital 1 NICU were stored with thioglycollate during the study period, identified between-site differences in these taxa were considered a potential artifact of the storage protocol, and analyses between sites were restricted to the taxa not influenced by storage protocol.

### Stool extraction and 16S rDNA sequencing

If stored in thioglycollate, stool was thawed and centrifuged for 10 min at 4,000 × *g* and the supernatant was removed. For all samples, 100 μL of TE buffer with lysozyme and proteinase K was added to 0.24 g of thawed stool and vortexed for 10 min. An amount of 1.2 mL of buffer RLT with beta-mercaptoethanol was added to the sample and transferred to sterile bead beating tubes containing 0.3 g of 0.1-mm glass beads. Samples were homogenized for 3 min in a bead beater and centrifuged at 4,000 × *g* for 5 min to pellet debris. Supernatant was transferred to a clean microcentrifuge tube and spun at 4,000 × *g* for an additional 2 min to remove remaining debris. Supernatant was then transferred to a Qiagen AllPrep DNA spin column, and DNA was isolated using the Qiagen AllPrep DNA/RNA mini kit (Qiagen, Valencia, CA, USA).

Using extracted DNA, 180-nt paired-end reads were generated using established primers and protocols, with samples allocated across multiple Illumina MiSeq (Illumina, San Diego, CA, USA) runs [14]. Read pairs were merged to create amplicon-spanning sequences that were then filtered to remove those with less than 70% identity to any read in the rRNA16S.gold.fasta reference set (http://drive5.com/uchime/uchime_download.html) using “usearch -usearch_global -id 0.70.” Using the UPARSE pipeline [15], software version usearch7.0.959_i86linux64, 79,076,883 sequences were processed. The following commands were used with default settings unless otherwise specified. Dereplication resulted in 35,605,130 sequences (-derep_fulllength); removal of singleton reads in 2,206,563 sequences (-sortbysize -minsize 2) and clustering yielded 7,249 OTU representative sequences (-cluster_otus). The OTU table was constructed by mapping reads to OTUs (-usearch_global -strand plus -id 0.97) and applying the python script uc2otutab.py (http://drive5.com/python/). Additional chimera filtering was not applied. QIIME [16] version 1.6 was used to provide classifications of the OTU representative sequences using the gg_13_5 GreenGenes taxonomy and representative sequences constructed at 99% similarity. A phylogenetic tree was constructed within the QIIME package using FastTree and filtered PyNAST alignments of the OTU representative sequences. OTUs with a minimum count fraction of 0.0002 were removed from the OTU table in QIIME, resulting in 525 unique OTUs.

### Statistical analysis

Comparisons between years and hospitals were restricted to week 1 and 2 samples, as hospital 2 had only a single sample from week 3 in 2011.

Differences in clinical characteristics among groups by week were tested using Fisher’s exact test for categorical variables and *t* test for continuous variables. To standardize comparisons of microbiota, we rarefied the OTU table to 2,000 reads per sample. Rarefaction randomly selects reads from the complete set obtained for each sample until the specified number of reads is obtained. This means that each sample has an equal chance of including rare OTUs so that the samples can be compared.

Alpha diversity was calculated for weeks 1 and 2 using two metrics: Simpson Diversity Index (1-D) and Chao1. Kruskal-Wallis (KW) was used to test for differences in alpha diversity by year and hospital. To examine beta diversity, we used non-metric dimensional scaling (NMDS) to ordinate the microbial communities based on both the unweighted and weighted UniFrac distance calculated in QIIME as described in Morrow et al. [10]. The unweighted UniFrac examines presence/absence only while the weighted UniFrac accounts for abundance differences.

Significant differences in specific taxa between hospitals and by year were identified by linear discriminant analysis effect size (LEfSe) [17]. GEE models were used assuming linear and logistic relationships to test the association of taxa identified by LEfSe with hospitals after adjustment with other potential confounders. A backwards elimination approach was used to remove non-significant covariates from the models. Samples from the same infant in different weeks were included in the same model.

Differences in degree of succession between hospitals were tested using the Jaccard index. Values for the Jaccard index were calculated between the weeks 1 and 3 samples from 28 infants who had samples collected across the first 3 weeks of life. The Jaccard index is a distance metric used to show similarity over time. Identical communities will have a Jaccard index value of 0 while completely non-overlapping communities will have a Jaccard index value of 1. KW was used to test for differences in the intra-subject Jaccard index values by hospital and year.

## Competing interests

The authors declare no competing interests.

## Authors’ contributions

ALM, KRS, DVW, and DSN designed the research. DHT and ALM analyzed the data. KRS and NA supervised the clinical research. ALM and DHT supervised sample collection and management. ZY and DHT conducted DNA extractions. DVW guided sequence data production and performed metagenomic analysis. DHT and ALM wrote the first draft and all authors contributed to writing or editing the manuscript.

## Supplementary Material

Additional files 1 **Metadata and metadata dictionary.** Metadata for all samples included in this is contained in the first tab, labelled Metadata. The second tab, labelled Data Dictionary, contains the data dictionary with definitions of all the variables included on the Metadata tab. Click here for file

Additional files 2 **Rarefied operational taxonomic unit table.** The first tab, labelled OTU table, contains the rarefied OTU table with data for each sample included in this study. The second tab, labelled Data Dictionary, contains information on the taxonomy of all OTUs in the OTU table. Click here for file
